# Evaluating the Implementation and Maintenance of a Breast Cancer Risk-Assessment and Prevention Program

**DOI:** 10.1245/s10434-025-18656-0

**Published:** 2025-11-01

**Authors:** Marybeth Hans, Arya S. Tamaskar, Abigail Recko, Brittany L. Bychkovsky, Lydia E. Pace, Tari A. King, Ko Un Park

**Affiliations:** 1 Division of Breast Surgery, Department of Surgery, Brigham and Women’s Hospital, Dana-Farber Cancer Institute, Boston, MA USA; 2 Breast Oncology Program, Dana-Farber Brigham Cancer Center, Boston, MA USA; 3 Division of Cancer Genetics and Prevention, Dana-Farber Cancer Institute, Boston, MA USA; 4 Harvard Medical School, Boston, MA USA; 5 Division of Women’s Health, Department of Medicine, Brigham and Women’s Hospital, Boston, MA USA; 6 Ariadne Labs, Brigham and Women’s Hospital, Harvard T.H. Chan School of Public Health, Boston, MA USA; 7 Present Address: Winship Cancer Institute of Emory University, Atlanta, GA USA

## Abstract

**Background:**

Understanding factors associated with successful high-risk breast health programs can aid in the development of similar initiatives. This study evaluated the impact of the Breast Cancer Personalized Risk Assessment, Education, and Prevention (B-PREP) program using the Reach, Effectiveness, Adoption, Implementation, and Maintenance (RE-AIM) framework.

**Methods:**

Patients evaluated from January 2017 to September 2024 were retrospectively reviewed from a prospectively maintained database. The number of patients seen over time was used to measure “reach.” Chemoprevention uptake was used to measure “effectiveness.” We surveyed B-PREP clinicians and staff using the validated Program Sustainability Assessment Tool (PSAT), to assess factors associated with sustainability (“implementation” and “maintenance”).

**Result:**

The study identified of 5972 B-PREP patients, 1860 (31.1 %) of whom had a high-risk lesion (HRL). The average time from the first visit to chemoprevention initiation was 57 weeks (median 24 weeks; range, 0–370 weeks). The overall chemoprevention initiation rate was 7.38 %, significantly higher for the patients with HRL (HRL [22.5 %] vs non- HRL [0.5 %]; *p* < 0.001). Sustained chemoprevention for ≥6 months also was higher with HRL (HRL [15.4 %] vs non-HRL [0.4 %]; *p* < 0.001). Ten stakeholders evaluated sustainability using the PSAT. The overall average was high (6.13 of 7). Domains on program adaptation (6.69), ongoing program evaluation (6.64), and environmental support (6.55) had the highest average.

**Conclusion:**

The comprehensive risk program increased annual chemoprevention use as clinic volume grew. As the program matured, time to chemoprevention initiation decreased. Clinicians noted that a supportive environment, regular program evaluation, and adaptation to changing circumstances were key features contributing to the sustainability of a successful high-risk program.

Individuals at elevated risk for the development of breast cancer have unique needs related to breast cancer surveillance, education, and risk management. Beginning with the publication of the NSABP P-1 trial in 1998, multiple studies have demonstrated the appropriateness of considering chemoprevention with selective estrogen receptor modulators or aromatase inhibitors for women at elevated risk for breast cancer.^1–4^ In their recommendation statements, The US Prevention Task Force, the National Comprehensive Cancer Network (NCCN), and the American Society of Clinical Oncology (ASCO) support this guidance as well.^5–7^

Several risk models have been developed to support clinicians in determining patients’ risk. These range from models relatively easy to incorporate into clinical workflow to models that are quite cumbersome,^8^ requiring clinicians to be aware of which model is most appropriate for the specific clinical scenario and making delivery by busy primary care practices challenging. Finally, options are increasingly available for imaging surveillance, which often require lengthy discussion regarding the pros and cons of each for a given individual. Given that chemoprevention and imaging have both benefits and risks, shared decision-making is needed, with extensive provision of patient education and consideration of patients’ values and preferences.^9–12^

Despite recommendations for tailored risk reduction and surveillance for patients at high risk for the development of breast cancer, a major gap exists between evidence-based guidelines and their implementation, with most patients at high risk for breast cancer not receiving recommended education or care. Moreover, the rates of chemoprevention uptake among high-risk individuals remain low, with a meta-analysis demonstrating an estimated pooled uptake of 15% to 16.3%.^13,14^ Health care systems have increasingly attempted to address this knowledge-implementation gap through developing clinics focused on care of those at elevated risk of breast cancer, but optimal strategies for delivery of this specialized care are not known.

One strategy used to deliver specialized care is through a comprehensive high-risk breast program. Establishing a high-risk breast program (also called “high-risk clinics” or “risk assessment and management programs”) can help identify, monitor, and manage the unique needs of patients with increased breast cancer risk due to genetic, familial, or personal factors.^15–18^ Specific considerations in designing a high-risk program include identifying the expected referral sources, ensuring reliable funding to adequately staff the program to allow for appropriate access, and providing ongoing educational support to clinicians staffing the program to ensure that patients receive consistent guideline appropriate care. Planning for ongoing program evaluation is critical to making process improvement changes where necessary and ensuring that the care provided is keeping pace with evolving data pertaining to surveillance and prevention options for this specialized patient population. Understanding the characteristics associated with the effectiveness (as measured by chemoprevention initiation rates) and sustainability of successful high-risk programs can assist in the development of similar initiatives.

This study aimed to identify and evaluate the impactful elements of the multidisciplinary, comprehensive, risk-assessment, and prevention program, B-PREP (Breast Cancer Personalized Risk Assessment, Education and Prevention), at Brigham and Women’s Hospital (BWH) in Boston, Massachusetts.

## Methods

### The B-PREP Program

The B-PREP program is nested within the Comprehensive Breast Health Center (CBHC) at BWH. Patients evaluated in the CBHC are invited for annual follow-up evaluation in the B-PREP program if they have received the diagnosis of a high-risk lesion (HRL), including atypical ductal hyperplasia (ADH), atypical lobular hyperplasia (ALH), and/or lobular carcinoma *in situ* (LCIS), or if they are estimated to be high risk for the development of breast cancer according to the Tyrer-Cuzick and/or Gail breast cancer risk models. High-risk patients without a high-risk lesion (non-HRL) must have a lifetime risk on the Tyrer-Cuzick model (either versions 7 or 8) of at least 20 % or a 5-year risk on the Gail model of at least 1.7 % (for patients ages 35 to 59 years) for inclusion in the B-PREP program. For patients age 60 years or older, a 5-year risk of at least 5 % was used for inclusion in the B-PREP program before 2019. However, with the subsequent use of low-dose tamoxifen with its favorable side-effect profile, lower 5-year risks were included as well.

Risk scores are generated using patients’ answers to a customized risk assessment survey, administered to all CBHC patients, which collects information about demographic, hereditary, reproductive, and clinical risk factors for breast cancer. Patients with a known or identified pathogenic genetic variant predisposing to elevated breast cancer risk are followed in the Cancer Genetics and Prevention Program at Dana-Farber Cancer Institute and thus were excluded from the current B-PREP program analysis. The overarching goals of the B-PREP program are to provide comprehensive breast cancer risk assessment, recommendations for tailored screening algorithms, and counseling on both pharmacologic and non-pharmacologic prevention strategies for the population of women evaluated in the CBHC.

The B-PREP program is staffed by a multi-disciplinary team consisting of specialty-trained physician assistants, medical oncologists, breast surgeons, and an internal medicine physician. Individualized counseling is augmented by robust patient educational materials, an annual free online forum addressing topics of interest to women at elevated risk, and a strategic focus on opportunities to participate in clinical trials focused on prevention. Although many women will choose not to pursue chemoprevention, a goal of the program is to ensure that women are making informed choices. Because many misconceptions are held by patients regarding medications for risk reduction,^13^ we believe increasing rates of chemoprevention uptake with decreasing time to intervention reflects the success of these educational efforts. We have previously demonstrated that supplemental screening MRI did not improve the cancer detection rate for patients with HRLs.^19^ Because the role of supplemental MRI is evolving in this population, use of supplemental MRI was not considered a key metric of success of the B-PREP program.

### Conceptual Model

We evaluated the implementation and impact of the B-PREP program using its Reach, Effectiveness, Adoption, Implementation, and Maintenance (RE-AIM) framework (Table 1).^20^ The five components of the RE-AIM framework can be used for planning and evaluation of a program.^21^ The RE-AIM domain definitions and key outcomes used to measure each domain are outlined in Table 1. *Reach* refers to the number and characteristics of individuals who participate in a program. It typically is calculated by comparing the characteristics of those who choose to participate with the characteristics of those who do not. *Effectiveness* or efficacy is the impact of the intervention, which we defined in this study as uptake of chemoprevention among high-risk patients. *Adoption* refers to the proportion and representativeness of settings and implementers who initiate a program. *Implementation* is the extent to which a program is delivered as intended. *Maintenance* is the extent to which a program is integrated into an institution and sustained over time.

**Table 1:** RE-AIM domain definitions and measurements used to evaluate the B-PREP program

RE-AIM domain	Definition and measurement
Reach	The absolute number of individuals willing to participate in B-PREP (representativeness of the B-PREP population previously reported).^20^ The number of patients evaluated in B-PREP as high risk for breast cancer
Effectiveness	The impact of the B-PREP program with respect to chemoprevention initiation. Number of patients taking chemoprevention
Adoption	The absolute proportion of patients identified as high-risk and referred to the B-PREP program relative to the broader eligible population of staff and settings. Previously reported^30^
Implementation	The degree to which the B-PREP program was implemented as intended. Program Sustainability Assessment Tool (PSAT) survey results
Maintenance	The degree to which the implementation of the evidence-based intervention was sustained as intended. Program Sustainability Assessment Tool (PSAT) survey results

### B-PREP Data

Patients evaluated in B-PREP from January 2017 to September 2024 were retrospectively reviewed from a prospectively maintained database. Information about the B-PREP population is stored in this database, including clinic visit dates, breast imaging and biopsies, use of risk-reducing medication, history of genetic testing, and development of primary breast cancer. These patients were sorted by HRL status (HRL vs non-HRL) and by year of first B-PREP visit.

Chemoprevention initiation (Effectiveness domain) and discontinuation among B-PREP patients was evaluated via chart abstraction. Chemoprevention initiation was limited to patients who started chemoprevention prescribed by a provider in the B-PREP program. Length of chemoprevention use was calculated only among regimens prescribed by B-PREP providers. Chemoprevention regimens included selective estrogen receptor modulators (raloxifene and tamoxifen) and aromatase inhibitors (exemestane, anastrozole, and letrozole). Tamoxifen was prescribed only at the full 20-mg dose until 2019, when the clinic began offering lose-dose (5-mg) tamoxifen for postmenopausal women based on the TAM-01 trial.^22,23^ Chemoprevention initiation included all the patients who started any B-PREP chemoprevention regimen for any length of time and was sorted by year of first regimen initiated. Time to initiation (TTI) of chemoprevention was calculated from patients’ first B-PREP visit and date of chemoprevention initiation.

### Sustainability Assessment

The Implementation and Maintenance domains of the RE-AIM framework were assessed using the previously validated Program Sustainability Assessment Tool (PSAT). This tool, developed in 2014 to aid in measuring a program’s capacity for sustainability, was designed to be easy to use as well as to be applicable for a variety of program types and sizes.^24^ The PSAT short version is a validated survey of three questions structured to evaluate eight domains using a 7-point Likert scale. Its domains are Environmental Support, Funding Stability, Partnerships, Organization Capacity, Program Evaluation, Program Adaptation, Communications, and Strategic Planning (Table 2).

**Table 2:** Program sustainability assessment tool (PSAT) domains, definitions, and examples

PSAT domains	Definition and examples
Environmental Support	Institution support; presence of a program “champion”
Funding Stability	Established consistent financial base for the program
Partnerships	Cultivation of connections between program and partners (breast imaging)
Organization Capacity	Integration of the program within the institution Division support–adequate staff to reach program goals
Program Evaluation	Regularly scheduled group meetings to discuss program performance Routine analysis of prospectively collected data
Program Adaptation	Implementation of changes in response to new science (low-dose tamoxifen) Before virtual risk assessments during COVID
Communications	Outreach to community PCP/GYN practices
Strategic Planning	Processes that guide program’s direction, goals, and strategies

PCP, primary care physician; GYN, gynecology

A purposive sample of 10 B-PREP clinicians, staff, administrators, and researchers was invited to participate. A facilitator (K.U.P.) introduced the PSAT and presented each participant with three survey questions per domain, allowing for a more accurate assessment of the domain’s impact. Individual scores per item and the average for each domain were ranked within a REDCap database. This was followed by using the Nominal Group Technique (NGT),^25^ whereby the individual items were discussed in a group, with each participant given the opportunity to explain the rationale for his responses, providing examples where applicable. The benefit of NGT is that through the structured discussion, it allows each stakeholder an equitable opportunity to contribute.

Responses then were discussed and ranked as a group, with the highest scores reflecting which domains were thought to represent the most impactful elements contributing to successful sustainability and maintenance of the program and the lowest scores reflecting where there was room for improvement. Because this analysis was part of a quality improvement study, the institutional review board approved it with exempt status.

## Results

### Reach and Effectiveness

**Fig. 1:**
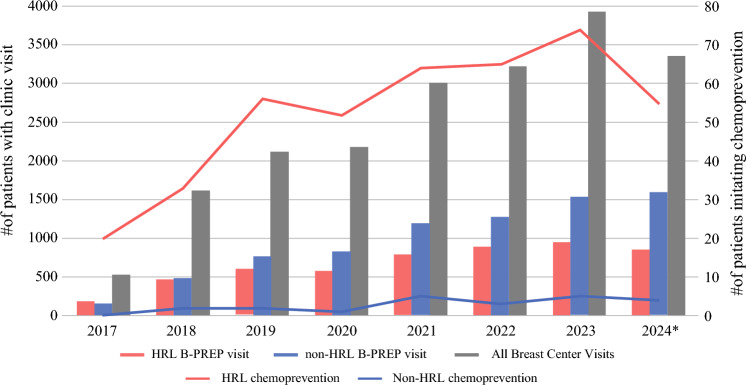
Annual B-PREP utilization (left axis, bar graph) and chemoprevention initiation (right axis, line graph) by high-risk lesion (HRL) status. *2024 Data are until September

Of the 5972 B-PREP patients identified in this study, 1860 (31.1 %) had a history of HRL. The median age was 46.3 years (range, 14.5–91.6 years), with 79.8 % identifying as white. The program demonstrated increased growth over time for both the patients with a history of a HRL (HRL B-PREP visit) and those at elevated risk without a history of a HRL (non-HRL B-PREP visit) (Fig. 1). The overall chemoprevention initiation rate was 7.38 %, significantly higher for the patients with an HRL (HRL [22.5 %] vs non-HRL [0.5 %]; *p* < 0.0001), particularly for the women older than 60 years. Sustained chemoprevention for 6 months or longer also was higher for those with an HRL (HRL [15.4 %] vs non-HRL [0.4 %]; *p* < 0.001).

The average time from first visit to chemoprevention initiation (TTI) was 57 weeks (median, 24 weeks; range, 0–370 weeks), and 14.9 % (*n* = 63) for the patients initiated within the first week after the initial consultation. The highest increase in chemoprevention initiation was in 2019, the year low-dose tamoxifen was adopted as an option in B-PREP.

### Sustainability Assessment

To assess maintenance of the RE-AIM framework, we used a validated PSAT survey. The participants in the NGT session were 10 stakeholders in roles representing clinical care (surgeons, medical oncologists, physician assistants, and nursing), administration (practice assistants, program manager), and research (clinical research coordinators). The overall PSAT average score was high (6.13), which aligns with the program’s long-term viability.

On a 7-point Likert scale, the only survey item that scored 7 (of 7) was regarding champions who support the program. Environmental Support, Program Evaluation, and Program Adaptation scored the highest average on the PSAT (Table 3). The key features representing Environmental Support were strong program champions and dedicated, passionate team members. Regularly scheduled group meetings to discuss program performance with routine analysis of prospectively collected data (Program Evaluation) was thought to be a key component of success. The core members of the B-PREP team, comprising surgeons, medical oncologists, an internist, physician assistants, nurses, and research assistants, meet bi-monthly to discuss new published research and emerging data.

**Table 3:** Program sustainability assessment tool (PSAT) score

Item	Environmental support	Funding	Partnerships	Organization capacity	Program evaluation	Program adaptation	Communication	Strategic planning
1	7	6.6	5.5	6.1	6.7	6.7	5.8	6.4
2	6.9	6	4.7	5.9	6.9	6.9	6.2	6
3	5.7	6.3	4.3	6.1	6.2	6.4	5.6	6.4
Average score	6.55	6.1	4.86	6.03	6.64	6.69	5.87	6.26

Cited examples of successful Program Adaptation were the program’s ability to pivot to virtual high-risk consultations during the COVID-19 pandemic (an option that continues to be chosen by many patients) and successful incorporation of the option of low-dose tamoxifen for chemoprevention in the postmenopausal setting after publication of the TAM-01 trial.^22,23^ In the group-ranking activity, Environmental Support was ranked highest, with Organizational Capacity and Program Adaptation ranked as the lowest contributors to sustainment of the program. Program Adaptation scored the highest average on the PSAT but ranked low during the group-ranking activity. This difference between the PSAT result and the group-ranking activity may be attributable to how the participants scored the survey questions in the Program Adaptation domain versus how they perceived the importance of Program Adaptation when taken comparatively against other domains.

The lowest score obtained was in the Partnership domain, indicating a need to strengthen collaborations both internally, mainly with breast imaging colleagues in the setting of constrained resources, and externally, with practices outside the institution to foster additional referral sources to enhance sustainability further.

## Discussion

Multiple randomized controlled trials have demonstrated the risk reduction benefits of chemoprevention for women at elevated risk for breast cancer.^1–4^ However, despite endorsement via established guidelines from ASCO,^6^ NCCN,^7^ and U.S. Preventive Services Task Force (USPSTF),^5^ implementing this practice through primary care providers has been unsuccessful largely because of perceived time constraints as well as an expressed lack of comfort in prescribing these medications.^26^ Given the complexity of individualizing prevention and surveillance recommendations, high-risk breast programs are well-positioned to provide the specialized services these women require.^27,28^

In our evaluation of the B-PREP program, we found that this initiative has successfully addressed the historic difficulties of translating evidence-based guidance into clinical care as demonstrated by increased chemoprevention use annually as the clinic volume grew, especially among patients older than 60 years or with HRL. As the program matured, the time to chemoprevention initiation decreased, likely reflecting efforts related to sustained provider engagement through ongoing dialogue and provision of robust patient education resources.

Although having a supportive champion was rated as the highest factor contributing to sustainability of the B-PREP program, there were other notable key features. Supportive environment, regular program evaluation, and adaptation to changing circumstances were key features contributing to the sustainability of a successful high-risk program. The wide range in time to chemoprevention initiation highlights the individualized nature of these decisions, and the relatively low rate (14.9 %) of chemoprevention initiation within the first week after initial consultation emphasizes the importance of fostering an ongoing patient-provider relationship to build on concepts outlined initially and to focus on any barriers that can be addressed by additional education. Multiple publications have reviewed processes to triage women for high-risk surveillance from mammography or primary care based on risk assessment surveys conducted in these settings.^16,18,29^ Some groups, including our own, have previously published on the structure of their high-risk programs^30^ or on specific clinical outcomes for their population.^31^ However, to date we are not aware of any published data leveraging implementation science methodologies to evaluate the effectiveness or sustainability of these programs.

The limitations of this study included the possibility that the assessed high-risk delivery model may not be generalizable to other settings, particularly those outside a large urban academic center. In particular, other high-risk programs also may include patients at high risk due to a pathogenic genetic variant, which the B-PREP does not manage because these patients are managed and followed in the Cancer Genetics and Prevention program. Furthermore, because chemoprevention initiation data was obtained through chart abstraction, missing or unclear provider documentation could have impacted our analyses.

In addition, because the clinic team was previously unfamiliar with the concepts around PSAT, the questions posed in the PSAT had varying interpretations, which may have affected the scores. However, during the NGT session, these issues were discussed and resolved during the group-ranking activity. For example, the Program Adaptation domain was one of the highest scoring domains in the PSAT but ranked low during the NGT session.

Finally, we chose to highlight the Effectiveness domain of RE-AIM by focusing on chemoprevention initiation and therefore did not fully evaluate the other domains. However, this could be addressed in future work.

In conclusion, the development and implementation of a high-risk breast cancer program such as B-PREP illustrates how to address the challenge of translating evidenced-based guidelines into real-world applications in a complex health care system. In this study, we leveraged several implementation science methodologies to evaluate the impact of the B-PREP program. Using the RE-AIM framework allowed us to focus on key domains for evaluation, specifically the reach and effectiveness of the program. The Program Sustainability Assessment Tool and use of the Nominal Group Technique provided a structure to critically evaluate which components of the program were key to its ongoing successful maintenance and which required additional attention for further improvement. Although more work remains to be done in terms of cultivating improved partnerships within the institution and surrounding communities, we demonstrated that supportive environment, regular program evaluation, and adaptation to changing circumstances were key features contributing to the sustainability of a successful high-risk program.

## Data Availability

The data will be made available to researchers whose proposed use of the data has been approved and after the completion of a signed data access agreement.
